# Comparison of short-term outcomes and defecatory function following robotic and conventional laparoscopic surgery for stapled-ileal pouch-anal anastomosis: a retrospective cohort study

**DOI:** 10.1097/JS9.0000000000001994

**Published:** 2024-07-24

**Authors:** Marie Hanaoka, Yusuke Kinugasa, Kenta Yao, Ayumi Takaoka, Megumi Sasaki, Shinichi Yamauchi, Masanori Tokunaga

**Affiliations:** Department of Gastrointestinal Surgery, Tokyo Medical and Dental University, Bunkyo-ku, Tokyo, Japan

**Keywords:** defecatory function, robotic surgery, stapled ileal pouch-anal anastomosis, short-term outcome, ulcerative colitis

## Abstract

**Background:**

This study assessed the potential advantages of robotic-assisted Stapled ileal pouch-anal anastomosis (Ro Stapled-IPAA) in ulcerative colitis (UC) compared to conventional laparoscopic surgery (Lap), with a focus on short-term outcomes and postoperative defecatory function, an aspect not previously explored.

**Materials and methods:**

Out of a total of 132 patients who underwent proctocolectomy or residual rectal resection, consecutive patients undergoing minimally invasive Stapled-IPAA for UC at our hospital from May 2014 to May 2024 were included. The Ro approach was chosen for individuals with severe colitis extending into the anal canal, deeper rectal cancers (beyond T1), and cases requiring residual rectal resection, taking advantage of its benefits. Perioperative outcomes, including anastomosis height, operative time, intraoperative blood loss, complication rate, postoperative hospital stay, and defecatory function using Wexner scores and anorectal manometry before proctocolectomy and 6 months after stoma closure, were compared between the Ro and Lap groups.

**Results:**

Thirty-three patients (Lap, *n*=21; Ro, *n*=12) were included. The Ro group demonstrated a significantly lower anastomosis height (0.5 vs. 3.0 cm, *P*<0.001), reduced intraoperative blood loss (35 vs. 118 ml, *P*=0.032), shorter postoperative hospital stay (8 vs. 10.5 days), and no cases of anastomotic leakage (0 vs. 14.3%), as compared to the Lap group. Pouch failure occurred in 14% of Lap group; none were observed in the Ro group. Wexner scores favored the Ro group at 12 months after stoma closure (0 vs. 8 points), and there was better maximum voluntary squeeze pressure (302 mmHg vs. 175 mmHg, *P*=0.03), indicating preserved contraction of the external sphincter muscle despite the lower anastomosis.

**Conclusion:**

Ro Stapled-IPAA for patients with UC led to better short-term outcomes and preservation of defecatory function with lower anastomosis than Lap, suggesting the clinical advantages of the robotic approach in this field.

## Introduction

Highlights

- The Robotic-Stapled-IPAA (Ro) group demonstrated superior outcomes with lower anastomosis, less intraoperative blood loss, reduced anastomotic leakage, and no pouch failures compared to Laparoscopic-Stapled-IPAA (Lap) group.
- The Wexner scores at 12 months after stoma closure favored the Ro group.
- Maximum voluntary squeeze pressure using intra-anal pressure assessments were superior in the Ro group.
- Favorable Wexner scores and better external anal sphincter function suggest the clinical advantages of the Ro approach.
- This study is the first to highlight the short-term and defecation function efficacy of the Ro approach in Stapled-IPAA compared to Laparoscopic-Stapled-IPAA.

Handsewn-ileal pouch-anal anastomosis (IPAA) or Stapled-IPAA are the standard procedures of choice for patients with ulcerative colitis (UC) with fulminant or severe, internal medical treatment-resistant disease or carcinoma due to inflammation. Although the rectal mucosal resection in Handsewn-IPAA reduces carcinogenic risks and minimizes complications, including rectal cuff enlargement^1^, postoperative tumor development from residual mucosa in Stapled-IPAA has been reported to be rare^2^, and Stapled IPAA has become widely used owing to its better postoperative bowel function, widespread stapled anastomosis, and stable technique^2^.

Minimally invasive IPAA surgery, especially laparoscopic procedures, has gained prominence^3^ and is currently widely performed, offering advantages such as reduced blood loss^4^, faster recovery^4^, enhanced fertility^5^, and improved aesthetics^6^ over open surgery. However, conventional laparoscopic surgery (Lap) for inflammatory bowel disease has limitations, including a high conversion rate to laparotomy (23.8%)^6^ and reduced operability in the deep pelvic region^1^ caused by forceps interference.

The emerging transanal total mesorectal excision technique for rectal cancer^7–9^ presents an alternative for minimally invasive IPAA; however, its unique challenges and learning curves should be considered^9^. The recently attempted robotic-assisted proctocolectomy for UC^1^ lacks substantial evidence supporting clinical superiority over laparoscopic procedures^1,10–15^. Additionally, the comparison of postoperative defecatory function between conventional laparoscopic Stapled-IPAA (Lap Stapled-IPAA) and robotic-assisted Stapled-IPAA (Ro Stapled-IPAA) remains unexplored.

Our hospital employs the Ro approach for specific indications, such as severe colitis into the anal canal and cases requiring residual rectal resection. Using precise manipulation in the deep pelvis, this study aimed to assess the short-term outcomes and postoperative defecatory function in patients undergoing Lap and Ro Stapled-IPAA.

## Methods

### Study design and patients

The protocol for this research project has been approved by the ethics committee of our university (approval no.: M2020-367; date of approval: 12th March 2021) and conforms to the provisions of the Declaration of Helsinki and registered withｊRCT. Written informed consent was obtained from all the participants. This study adhered to the Strengthening the reporting of cohort, cross-sectional and case–control studies in surgery (STRCOSS, Supplemental Digital Content 1, http://links.lww.com/JS9/D179) guidelines^16^.

**Figure 1:**
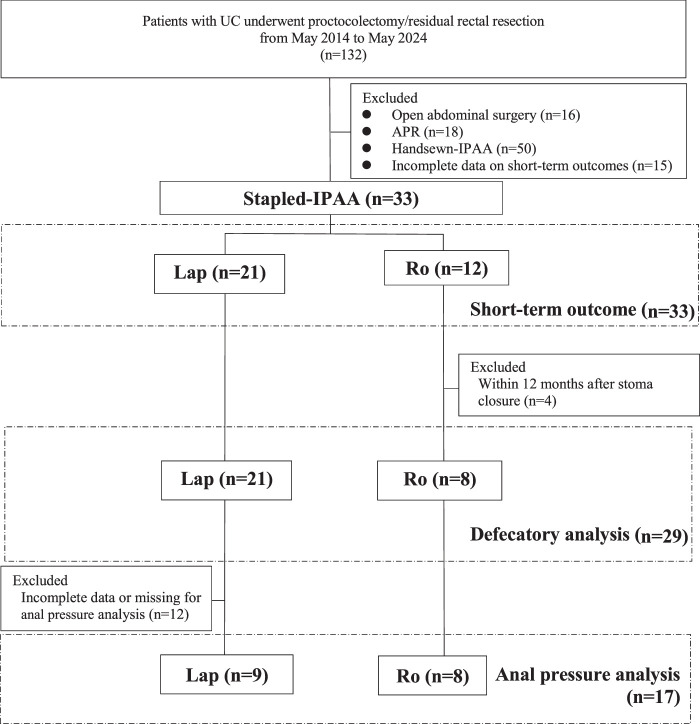
Flow diagram of the study. APR, abdominal perineal resection; IPAA, ileal pouch-anal anastomosis; Lap, conventional laparoscopic surgery; Ro, robotic-assisted surgery; UC, ulcerative colitis.

We retrospectively investigated 132 consecutive patients with UC who underwent proctocolectomy or residual rectal resection between May 2014 to May 2024 at our hospital. Patients undergoing open abdominal surgery, abdominoperineal resection (APR), and Handsewn-IPAA were excluded (Fig. 1). Patients were categorized into the following two groups based on surgical strategy: Lap and Ro groups.

The selection between the Ro and Lap approach in Stapled-IPAA was determined during a preoperative conference within the department. Ro Stapled-IPAA allows precise manipulation within the pelvis and deep rectal mobilization up to the anal canal. In this regard, Ro Stapled-IPAA was selected for cases with severe colitis extending to the anal canal, deeper rectal cancers (beyond T1), or residual rectal resection in the second stage of a three-stage surgery predominantly involving intrapelvic manipulation.

### Standard surgical protocol

Our standard protocol involved a two-stage surgery for UC cases, comprising Stapled-IPAA or Handsewn-IPAA in the first stage and stoma closure in the second stage. The decision between Handsewn-IPAA and Stapled-IPAA, made by the attending surgeon before July 2017, shifted to a principle of transanal minimally invasive proctocolectomy (TAMIP) from August 2017 onward. This decision aimed to ensure reliable mucosa evacuation of the anal canal and shorter operative time performed using the TAMIP approach. Stapled-IPAA was preferred in cases where there was a risk of the ileal pouch not reaching the anus, generally determined by factors of tall stature, narrow pelvis, diffuse adhesions^17,18^, and high BMI^19,20^. A BMI ≥25 kg/m^2^ was set as the cutoff for Stapled-IPAA based on past experiences in our institution. Reconstruction methods in cases with BMI ≥25 kg/m^2^ and <25 kg/m^2^ were Stapled-IPAA and Handsewn-IPAA, respectively.

In Ro Stapled-IPAA, we exclusively employed the da Vinci Xi system (Intuitive Surgical). The data for this study were accumulated from 2014 onwards, but all robotic procedures were performed using the da Vinci Xi Surgical System. The reason for this choice is that our hospital began performing Ro Stapled-IPAA around the same time that robotic-assisted rectal surgery became covered by insurance in April 2018 in Japan. While our hospital possesses both the da Vinci Xi and X models, we opt for the da Vinci Xi when performing total proctocolectomy to accommodate a wider surgical field.

### Three-stage operation for fulminant cases

For fulminant and severe cases, a three-stage operation was selected, involving subtotal colorectal resection in the first stage, residual rectal resection with Stapled-IPAA or Handsewn-IPAA in the second stage, and stoma closure in the third stage. Cases with highly risky or undesired anastomosis underwent proctocolectomy with abdominal perineal resection (APR) from the beginning.

### Stapled-IPAA approaches

Stapled-IPAA included Lap and Ro approaches. The Ro approach, introduced in November 2021, excels in precise pelvic manipulation and is indicated for patients with severe colitis extending into the anal canal, rectal cancer deeper than T1, and cases of residual rectal resection in the second stage of a three-stage operation.

### Robotic-assisted proctocolectomy

**Figure 2:**
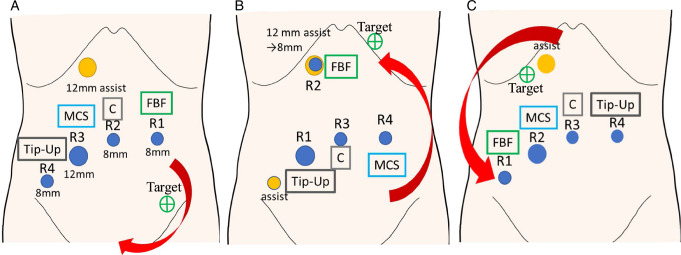
Port placement of robotic total proctocolectomy. (A) Mobilization of the rectum. (B) Mobilization of the splenic fracture and left side of the transverse colon. (C) Mobilization of the right side of the transverse and ascending colon. R1: arm 1 of the da Vinci Xi; R2: arm 2 of the da Vinci Xi; R3: arm 3 of the da Vinci Xi; R4: arm 4 of the da Vinci Xi. C, camera; MCS, monopolar curved scissors; FBF, fenestrated bipolar forceps; Tip-Up, tip-up fenestrated grasper.

Using the da Vinci Xi surgical system with three 8 mm robotic ports, one 12 mm robotic port, and one 12 mm auxiliary port for the assistant (Fig. 2), Ro proctocolectomy was performed. The da Vinci port was inserted at the umbilical site, and additional ports were strategically placed on a 45° left oblique upward line for optimal pelvic manipulation (Fig. 2a), following a medial approach similar to low anterior resection for rectal cancer^21^. The procedure began with a medial-to-lateral approach and the dissection of the inferior mesenteric artery and inferior mesenteric vein, followed by descending colon mobilization. Mobilization extended up to the anal canal. Subsequent steps included the mobilization of the splenic flexure and transverse colon. In a port-in-port configuration, the da Vinci 8 mm port was inserted into the 12 mm assistant port, precisely targeting the splenic flexure (Fig. 2b). This facilitated the mobilization of the splenic flexure and the left side of the transverse colon. The mobilization of the right side of the transverse colon extended to the hepatic flexure, completing the mobilization of the entire right colon. For patients without colon cancer, the ileocecal artery was preserved, and the mesentery was excised from the right side of the colon to the rectum close to the intestinal tract. In cases involving cancer, the ileocecal artery was managed as necessary, and lymph nodes were dissected. Rectal dissection in the anal canal was typically accomplished using a 45 mm da Vinci Stapler, commonly employing a green cartridge. Employing Alexis wound protection (Applied Medical), the incision of the umbilical port wound was extended to 4–5 cm. Subsequently, an extracorporeal creation of an 18–20 cm long ileal pouch was performed after specimen removal. The ileoanal anastomosis was established using a double stapling technique with a 25 mm circular stapler. Finally, a temporary ileal double-barrel ostomy was created in the right lower abdomen. As a rule, the interval between the second and third stages (stoma closure) in the three-stage surgery and the first and second stages (stoma closure) in the two-stage surgery was set to 3 months, with no cases exceeding 12 months.

### Robotic-assisted residual rectal resection

In the Ro residual rectal resection, the procedure involved resecting the remaining rectum, establishing ileoanal anastomosis, and creating a temporary ileostomy in patients who had previously undergone an emergency subtotal colectomy ~3 months earlier. The techniques for rectal mobilization, anastomosis, and temporary ileostomy were consistent with those used in proctocolectomy and are not detailed here.

### Conventional laparoscopic proctocolectomy and residual rectal resection

For Lap proctocolectomy and residual rectal resection, a 12 mm camera port was inserted at the umbilicus, a 12 mm port at the right lower abdomen, and 5 mm ports at the right abdomen, left lower abdomen, and left abdomen. In some cases, an additional 5 mm port was added to the right upper abdomen during splenic flexure mobilization, depending on the body shape. The procedure closely resembled Ro proctocolectomy and residual rectal resection. Rectal mobilization was performed as extensively as possible towards the anorectal side, and the rectum was dissected using laparoscopic staplers.

### Evaluation of short-term outcome

The evaluation of short-term outcomes involved the collection of data from medical records on age, sex, BMI, American Society of Anesthesiologists physical status (ASA-PS), duration of UC, type of surgery, reason for the surgery, preoperative serum albumin, rate of conversion to laparotomy, operative time, blood loss, anastomosis height from the anal verge (AV), postoperative complications (defined as anastomotic or suture leakage, bleeding, small bowel obstruction, paucities, and others), postoperative hospital stay, rate of readmission within 30 days, rate of pouch failure, and rate of mortality. Pouch failure was defined as the need for pouch resection, permanent diversion, or a redo pouch^22^. Postoperative complications were categorized and assessed using the Clavien–Dindo classification^23^. Short-term outcomes for patients undergoing the 3-stage surgery were based on data from the second stage (residual rectal resection and Stapled-IPAA) rather than the first stage (subtotal colectomy).

### Evaluation of defecatory function

Patients undergoing Handsewn or Stapled-IPAA participated in a prospective survey for defecatory function questionnaire and intra-anal pressure measurement after providing informed consent.

For the questionnaire on defecatory function, at 6 and 12 months poststoma closure, patients voluntarily submitted questionnaires on defecatory function to the outpatient secretary, in the absence of medical stuffs. The questionnaire included the Cleveland Clinic Florida-Fecal Incontinence Score (Wexner score), bowel movement frequency, and original survey questionnaire, including the frequency of ‘soiling’, which was defined as a condition where a stain of 3 cm or more in diameter was observed at least three times per week, and ‘spotting’ as a smaller stain^24^.

Intra-anal pressure measurements were performed before proctocolectomy and after 6 months from stoma closure. This study used a High-resolution Anorectal Manometry catheter with 40 pressure-sensing channels organized into sets of four circumferential and longitudinal channels. The manometric and topographic data were visualized on a computer monitor through specialized software (HR-Anorect.dll, Star Medical). Bowel preparation was not routinely performed. Patients were positioned in the left lateral decubitus with knees and hips flexed at a 90° angle, and the lubricated probe was gently inserted into the anal canal. Once properly situated, the probe assembly remained fixed throughout the study. The procedure included an assessment of functional anal canal length (FACL), maximum resting pressure (MRP), atmospheric maximum voluntary squeeze pressure (aMSP), and incremental maximum voluntary squeeze pressure (iMSP). For preoperative intra-anal pressure measurement, patients who required emergency surgery or presented with severe diarrhea or significant rectal bleeding due to the condition of UC were excluded.

### Statistical analysis

Categorical variables are presented as numerical values (%), and continuous variables are expressed as median and range. To assess the significance of differences between groups, Fisher’s exact and Mann–Whitney’s *U* tests were used for categorical and continuous variables, respectively. All statistical analyses were performed using EZR (The R Foundation for Statistical Computing), a graphical user interface for R^25^. Statistical analysis was performed by MH, who has statistical expertise, with the support of a statistician.

## Results

Figure 1 shows the patient selection flowchart of this study. Among 132 patients with UC who underwent proctocolectomy/residual rectal resection, 16 patients who underwent open surgery, 18 cases of APR, 50 cases of Handsewn-IPAA, and 15 cases with incomplete data on short-term outcomes were excluded from this study. Finally, 33 patients who underwent Stapled-IPAA were retained and categorized into Lap and Ro groups.

Table 1 outlines the patient background of both groups. The Lap group comprised 21 patients (15 and 6 with 2-staged and 3-staged surgery, respectively), and the Ro group had 12 patients (9 and 3 with 2-staged and 3-staged surgery, respectively). No significant differences were observed in age, sex, or BMI between the groups. The Ro group had a higher prevalence of UC-associated neoplasia (colorectal cancer or high-grade dysplasia) (Lap vs. Ro; 9.5 vs. 50%, *P*=0.015).

**Table 1:** Comparison of patient background between Ro vs. Lap in Stapled-IPAA.

Characteristics	Lap group *n*=21	Ro group *n*=12	*P*
Age (years)^a^	38 (17–73)	46.5 (28–68)	0.203
Sex (male)	15 (71)	12 (100)	0.065
BMI (kg/m^2^)^a^	20 (15–33)	22 (19–28)	0.120
ASA-PS (I/II/III)	1/16/4	0/11/1	0.762
Serum albumin (g/dl)^a^	2.8 (1.5–4.1)	4.2 (4.0–4.6)	0.076
Duration of UC (years)^a^	6.5 (0–33)	8 (0–37)	0.068
Emergency operation	4 (19.0)	0	0.271
Reason for the surgery			
UCAN or dysplasia	2 (9.5)	6 (66.7)	0.015
Resistance to treatment	19 (90.5)	6 (33.3)	
2-staged or 3-staged surgery			
2-staged	15 (71.4)	9 (75.0)	1.000
3-staged	6 (28.6)	3 (25.0)	

a Median, range.

ASA-PS, American Society of Anesthesiologists physical status; IPAA, ileal pouch-anal anastomosis; Lap, conventional laparoscopic; Ro, robotic-assisted surgery; UC, ulcerative colitis; UCAN, ulcerative colitis-associated neoplasia.

Short-term outcomes are presented in Table 2. The Ro group exhibited significantly lower blood loss (118 vs. 35 ml, *P*=0.032) and no anastomotic/suture leakage, whereas the Lap group had a leakage rate of 14.3%. Postoperative hospital stay tended to be shorter in the Ro group (10.5 vs. 8 days). No significant differences in operative time, postoperative complication rate (Clavien–Dindo classification grade ≥II), or readmission within 30 days were observed. The anastomosis height from the AV was significantly lower in the Ro group (3.0 vs. 0.5 cm, *P*<0.001). Pouch failure occurred in 14.3% of individuals in the Lap group and none in the Ro group. All patients who experienced pouch failure underwent creating permanent ileostomy.

**Table 2:** Comparison of short-term outcomes between Ro vs. Lap in Stapled-IPAA.

Characteristics	Lap *n*=21	Ro *n*=12	*P*
Conversion to laparotomy	0	0	1.000
Operative time (min)^a^	377 (210–714)	412 (251–559)	0.671
Blood loss (ml)^a^	118 (12–1164)	35 (0–190)	0.032
Anastomosis AV (cm)^a^	3.0 (0.5–8.0)	0.5 (0.5–3.0)	<0.001
Postoperative complication^b^ CD grade ≥II	9 (42.9)	5 (41.7)	1.000
CD II/III	5/4	4/1	—
Anastomotic/suture leakage	3 (14.3)	0	0.284
Small bowel obstruction	4 (19.0)	3 (25.0)	0.686
Bleeding	0	0	0
Paucities	2 (9.5)	2 (16.7)	0.610
Others	0	0	0
Postoperative hospital stay^a^	10.5 (6–39)	8 (6–34)	0.133
Readmission within 30 days	1 (4.8)	1 (8.3)	1.000
Pouch failure	3 (14.3)	0	0.284
Mortality	0	0	1.000

a Median, range.

b Within 30 days postoperatively.

AV, anal verge; CD, Clavien–Dindo classification; IPAA, ileal pouch-anal anastomosis; Lap, conventional laparoscopic; Ro, robotic-assisted.

**Figure 3:**
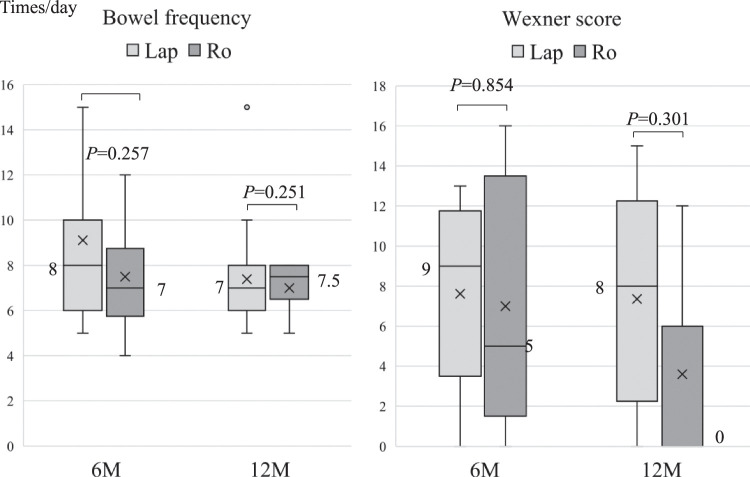
Comparison of postoperative trends in defecatory frequency and Wexner score. Boxes indicate interquartile ranges. Lines are the median, whereas bars are the range of scores. Mean values are indicated by a cross inside the box plot. Lap, conventional laparoscopic surgery; Ro, robot-assisted surgery.

Postoperative defecatory function results are shown in Figure 3. At 6 months postoperatively, the median frequency of bowel movements was 8 vs. 7 times/day in each group (*P*=0.257). At 12 months, the median frequency of bowel movements was 7 vs. 7.5 times/day in each group (*P*=0.251). The Wexner score at 6 months showed a negligible difference with no significant difference, while at 12 months, the Ro group outperformed the Lap group (8 vs. 0 points) (*P*=0.301). The breakdown of the Wexner score and result of the original survey questionnaire is shown in Supplemental Fig. 1 (Supplemental Digital Content 2, http://links.lww.com/JS9/D180).

**Figure 4:**
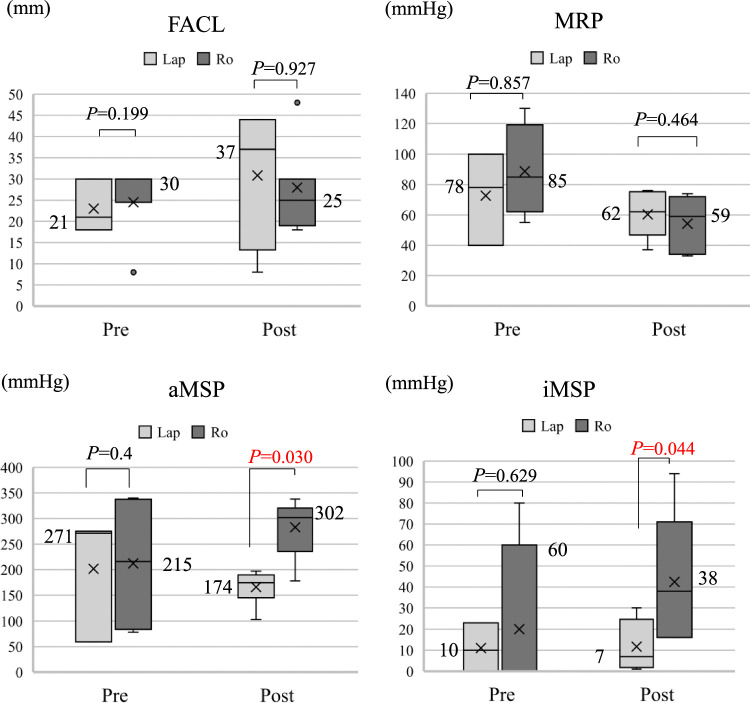
Comparison of preoperative and postoperative intra-anal pressure measurement between Lap and Ro groups. Boxes indicate interquartile ranges. Lines are the median, bars are the range of scores, and the X mark is the average value. FACL, functional anal canal length; MRP, maximum resting pressure; aMSP, atmospheric maximum voluntary squeeze pressure; iMSP, incremental maximum voluntary squeeze pressure.

In an investigation comprising 17 patients (Lap, *n*=9; Ro, *n*=8) who underwent intra-anal pressure assessments, four parameters, including FACL, MRP, aMSP, and iMSP, were measured before proctocolectomy and 6 months poststoma closure (Fig. 4). No significant differences were observed in patient characteristics, including age, sex, BMI, ASA-PS, duration of UC, reason of the surgery, and type of surgery between the groups (data not shown). Regarding FACL, there was no significant difference between the groups preoperatively. Although it was slightly better in the Lap group (37 mm vs. 25 mm) postoperatively, the difference was not significant. For MRP, the preoperative values were slightly higher in the Ro group (78 mmHg vs. 85 mmHg), but postoperative values were comparable between the two groups. For aMSP, the preoperative values were slightly higher in the Lap group, but postoperative values were significantly higher in Ro group (302 mmHg) than the Lap group (175 mmHg) (*P*=0.030). Similarly, for iMSP, the postoperative outcomes were significantly better in the Ro group compared to the Lap group (*P*=0.044).

## Discussion

In this study, the short-term outcomes and postoperative defecatory function were compared between Lap and Ro approaches for Stapled-IPAA in patients with UC. The Ro group demonstrated superior outcomes with lower anastomosis, less intraoperative blood loss, reduced anastomotic leakage, and no pouch failures. The Wexner scores at 12 months after stoma closure favored the Ro group. Furthermore, iMSP and aMSP using intra-anal pressure assessments, which reflected external anal sphincter function, were superior in the Ro group, indicating preserved defecatory function despite the lower anastomosis. This study is the first to highlight the short-term efficacy of the Ro approach in Stapled-IPAA and to compare the defecatory function between Ro and Lap Stapled-IPAA.

Although evidence on the efficacy of Ro Stapled-IPAA is limited, it has been reported that the Ro approach enables deeper pelvic access^1^ and suggested to reduce pouch failure risk by achieving a low anastomosis^1^. Our findings align with these trends, as the Ro group exhibited significantly lower anastomosis (3.0 cm in Lap vs. 0.5 cm in Ro group) and zero pouch failures than the Lap group.

Concerning anastomotic safety, the Ro approach has been associated with reduced anastomotic leakage due to improved staple manipulation^26^. Our results supported this, with no cases of anastomotic leakage in the Ro group compared to 14.3% in the Lap group. Additionally, consistent with the previous report^27^, the Ro group showed comparable operative times and significantly shorter postoperative hospital stay, which could be potentially attributed to fewer anastomotic leakages.

Limited studies exist on the benefits of the Ro approach with respect to functional evaluation^11,12^, and no reports have compared the defecatory frequency or postoperative defecatory function between Ro and Lap Stapled-IPAA^1,28^. In this study, the Ro group demonstrated a better Wexner score at 12 months postoperatively (8 vs. 0 points, Lap vs. Ro, respectively).

Anorectal manometry, which provides a dynamic measure of intraluminal pressure, is the best established and most widely available investigative tool in the diagnostic armamentarium and enables an objective evaluation of parameters of both anal and rectal function, such as tone, contractility, and relaxation^29^. FACL is the length of the anal canal as measured using anorectal manometry, and longer FACL is generally considered more favorable for stool incontinence. MRP generally reflects the force of contraction of the internal anal sphincter muscle. aMSP reflects the force of contraction of both the puborectalis muscle and the external anal sphincter function; however, iMSP reflects external anal sphincter function^30^.

The external anal sphincter is innervated by the fourth sacral, anococcygeal, inferior rectal, and perineal nerves, all of which branch from the pudendal and caudal plexus^31^. Given these, the positive iMSP and aMSP in the Ro group, which showed better external anal sphincter function, could be attributed to the potential for preserving these nerves and the external anal sphincter itself^32^.

Although the correlation between anal pressure measurements and fecal incontinence remains debatable, previous studies suggest that decreased MRP is associated with leaky fecal incontinence^33,34^, and decreased MSP is linked to urge incontinence^33^. Despite the Ro group having a significantly lower anastomosis, its internal anal sphincter function (MRP) was slightly lower than that of the Lap group but maintained, while external anal sphincter function (MSP) was highly preserved. Postoperative Wexner scores in the Ro group in postoperative 12 months showed a trend toward superiority, which was linked to the superiority of MSP and generally leads to superiority in urge incontinence. However, since the Wexner score does not comprehensively assess fecal incontinence details (such as incontinence or urgency), a more detailed study using an alternative index would be preferable.

This study has some limitations, particularly its single-center retrospective design, potential selection bias, and small sample size, which may limit the statistical power. The small number of patients in this study, which focus on patients underwent Stapled-IPAA can be attributed to the fact that complete anal mucosal resection with Handsewn-IPAA was more widely performed as the most effective treatment. Additionally, the limited number of Ro approach is due to the recent approval of insurance coverage for robot-assisted rectal and colorectal surgeries in Japan, and the very few facilities performing robot-assisted proctocolectomies for UC. This factor have made multicenter studies challenging.

During the evaluation of postoperative anorectal function, a potential bias emerged in anastomosis height comparisons between groups, with a preference observed in favor of the Lap group. Notwithstanding this bias, the collective postoperative anorectal function demonstrated superiority in the Ro group. These findings imply a greater potential for enhanced postoperative anorectal function with the Ro approach, even in the presence of the observed bias.

Next, regarding anal pressure measurements, as mentioned in the Methods section, we excluded cases involving emergency surgeries, patients in poor condition, and those with severe diarrhea or frequent rectal bleeding before proctocolectomy. Additionally, although measurements could be taken, some patient’s anal pressure measurements could not be accurately assessed due to the symptoms of UC. Consequently, it is possible that some patients were considered to have worse anorectal function preoperatively compared to their postoperative status. Moreover, the short observation period for intra-anal pressure measurements further hinder comprehensive comparisons. Additionally, the timing of intra-anal pressure measurements varied from 6 months onward. Therefore, future studies should address these limitations through larger multicenter trials, and extended follow-up to validate these findings.

Despite these limitations, this study is the first to report on the short-term efficacy of the Ro approach in Stapled-IPAA for UC, comparing it with the Lap approach. Further validation through increased case accumulation is necessary for a comprehensive understanding, particularly in patients with rectal cancer or obesity, for whom the Ro approach may offer advantages in maneuverability in the deep pelvic region.

## Conclusion

Ro Stapled-IPAA for patients with UC demonstrated superior short-term outcomes, including lower anastomosis height, intraoperative blood loss, shorter hospital stay, and decreased anastomotic leakage and pouch failure. Favorable Wexner scores and better external anal sphincter function suggest the clinical advantages of the Robotic-assisted approach over conventional laparoscopic approach in this context.

## Ethical approval

The protocol for this research project has been approved by the Ethics Committee of Tokyo Medical and Dental University (approval no.: M2020-367) and conforms to the provisions of the Declaration of Helsinki.

## Consent

Written informed consent was obtained from all patients.

## Source of funding

This research was supported by Takeda Japan Medical Office Funded Research Grant 2022.

## Author contribution

M.H.: designed the study, provided the main conceptual ideas, and proofread the outline; Y.K., M.T., and S.Y.: made critical revisions; K.Y., A.T, M.S., and S.Y.: collected the data; M.T. and Y.K.: provided the final approval for the manuscript. All authors read and approved the final manuscript.

## Conflicts of interest disclosure

Yusuke Kinugasa received speaker honoraria from Intuitive Surgical, Johnson & Johnson KK, and Medtronic Japan. Masanori Tokunaga received speaker honoraria from Johnson & Johnson KK, Medtronic Japan, Olympus, and Intuitive Surgical. The other authors have no conflicts of interest, funding, or other sources of support to declare in relation to the submitted article.

## Research registration unique identifying number (UIN)

1. Name of the registry: jRCT.
2. Unique identifying number or registration ID: jRCT1030230639.
3. Hyperlink to your specific registration (must be publicly accessible and will be checked): https://jrct.niph.go.jp/re/reports/detail/78292.

## Guarantor

Prof. Yusuke Kinugasa, Professor of Department of Gastrointestinal Surgery, Tokyo Medical and Dental University, Tokyo, Japan.

## Data availability statement

We confirm that the datasets generated and/or analyzed during the current study are available upon reasonable request. Further details regarding data availability can be provided upon request to the corresponding author.

## Provenance and peer review

Not commissioned, externally peer-reviewed.

## Presentation

Presentation by: Marie Hanaoka, Ayumi Takaoka, Megumi Sasaki, Shinichi Yamauchi, Yusuke Kinugasa.

Affiliation: Department of Gastrointestinal Surgery, Tokyo Medical and Dental University, Tokyo, Japan.

Date: 27th September 2023.

Venue: 18th Scientific Conference, European Society of Coloproctology (ESCP), held in Vilnius, Lithuania.

Presentation Format: Poster Presentation.

## Supplementary Material

## References

[1] Lynch AC . Robotic surgery for the ileal pouch. Dis Colon Rectum 2022;65:S37–S40. 35867639 10.1097/DCR.0000000000002549

[2] Kuwabara H Kimura H Kunisaki R . Postoperative complications, bowel function, and prognosis in restorative proctocolectomy for ulcerative colitis—a single-center observational study of 320 patients. Int J Colorectal Dis 2022;37:563–572. 34751417 10.1007/s00384-021-04059-6

[3] Antolovic D Kienle P Knaebel HP . Totally laparoscopic versus conventional ileoanal pouch procedure–design of a single-centre, expertise based randomised controlled trial to compare the laparoscopic and conventional surgical approach in patients undergoing primary elective restorative proctocolectomy–LapConPouch-Trial. BMC Surg 2006;6:13. 17125500 10.1186/1471-2482-6-13 PMC1676020

[4] Nozawa H Hata K Sasaki K . Laparoscopic vs open restorative proctectomy after total abdominal colectomy for ulcerative colitis or familial adenomatous polyposis. Langenbecks Arch Surg 2022;407:1605–1612. 35294600 10.1007/s00423-022-02492-x

[5] Bartels SA DʼHoore A Cuesta MA . Significantly increased pregnancy rates after laparoscopic restorative proctocolectomy: a cross-sectional study. Ann Surg 2012;256:1045–1048. 22609840 10.1097/SLA.0b013e318250caa9

[6] Schiessling S Leowardi C Kienle P . Laparoscopic versus conventional ileoanal pouch procedure in patients undergoing elective restorative proctocolectomy (LapConPouch Trial)–a randomized controlled trial. Langenbecks Arch Surg 2013;398:807–816. 23686277 10.1007/s00423-013-1088-z

[7] Coffey JC Dillon MF O’Driscoll JS . Transanal total mesocolic excision (taTME) as part of ileoanal pouch formation in ulcerative colitis–first report of a case. Int J Colorectal Dis 2016;31:735–736. 26033482 10.1007/s00384-015-2236-4

[8] de Buck van Overstraeten A Mark-Christensen A Wasmann KA . Transanal versus transabdominal minimally invasive (completion) proctectomy with ileal pouch-anal anastomosis in ulcerative colitis: a comparative study. Ann Surg 2017;266:878–883. 28742696 10.1097/SLA.0000000000002395

[9] Park L Truong A Zaghiyan K . A single-center comparative study of open transabdominal and laparoscopic transanal ileal pouch-anal anastomosis with total mesorectal excision. Has the bar been raised. J Gastrointest Surg 2022;26:1070–1076. 34993896 10.1007/s11605-021-05236-2

[10] Panteleimonitis S Al-Dhaheri M Harper M . Short-term outcomes in robotic vs laparoscopic ileal pouch-anal anastomosis surgery: a propensity score match study. Langenbecks Arch Surg 2023;408:175. 37140753 10.1007/s00423-023-02898-1 PMC10160174

[11] Miller AT Berian JR Rubin M . Robotic-assisted proctectomy for inflammatory bowel disease: a case-matched comparison of laparoscopic and robotic technique. J Gastrointest Surg 2012;16:587–594. 21964583 10.1007/s11605-011-1692-6

[12] Rencuzogullari A Gorgun E Costedio M . Case-matched comparison of robotic versus laparoscopic proctectomy for inflammatory bowel disease. Surg Laparosc Endosc Percutan Tech 2016;26:e37–e40. 27258914 10.1097/SLE.0000000000000269

[13] Lightner AL Grass F McKenna NP . Short-term postoperative outcomes following robotic versus laparoscopic ileal pouch-anal anastomosis are equivalent. Tech Coloproctol 2019;23:259–266. 30941619 10.1007/s10151-019-01953-8

[14] Mark-Christensen A Pachler FR Nørager CB . Short-term outcome of robot-assisted and open IPAA: an observational single-center study. Dis Colon Rectum 2016;59:201–207. 26855394 10.1097/DCR.0000000000000540

[15] Flynn J Larach JT Kong JCH . Robotic versus laparoscopic ileal pouch-anal anastomosis (IPAA): a systematic review and meta-analysis. Int J Colorectal Dis 2021;36:1345–1356. 33611619 10.1007/s00384-021-03868-z

[16] Mathew G Agha R for the STROCSS Group . STROCSS 2021: strengthening the reporting of cohort, cross-sectional and case-control studies in surgery. Int J Surg 2021;96:106165. 34774726 10.1016/j.ijsu.2021.106165

[17] Smith L Friend WG Medwell SJ . The superior mesenteric artery. The critical factor in the pouch pull-through procedure. Dis Colon Rectum 1984;27:741–744. 6499610 10.1007/BF02554606

[18] İsmail E Açar Hİ Arslan MN . Comparison of mesenteric lengthening techniques in IPAA: an anatomic and angiographic study on fresh cadavers. Dis Colon Rectum 2018;61:979–987. 29994960 10.1097/DCR.0000000000001133

[19] Khasawneh MA McKenna NP Abdelsattar ZM . Impact of BMI on ability to successfully create an IPAA. Dis Colon Rectum 2016;59:1034–1038. 27749478 10.1097/DCR.0000000000000686

[20] Poh KS Qureshi S Hong YK . Multivariate prediction of intraoperative abandonment of ileal pouch anal anastomosis. Dis Colon Rectum 2020;63:639–645. 32032200 10.1097/DCR.0000000000001617

[21] Shiomi A Kinugasa Y Yamaguchi T . Robot-assisted rectal cancer surgery: short-term outcomes for 113 consecutive patients. Int J Colorectal Dis 2014;29:1105–1111. 24942499 10.1007/s00384-014-1921-z

[22] Alsafi Z Snell A Segal JP . Prevalence of ‘pouch failure’ of the ileoanal pouch in ulcerative colitis: a systematic review and meta-analysis. Int J Colorectal Dis 2022;37:357–364. 34825957 10.1007/s00384-021-04067-6 PMC8803821

[23] Dindo D Demartines N Clavien PA . Classification of surgical complications: a new proposal with evaluation in a cohort of 6336 patients and results of a survey. Ann Surg 2004;240:205–213. 15273542 10.1097/01.sla.0000133083.54934.ae PMC1360123

[24] Futatsuki R Sugita A Koganei K . Clinical analysis of the postoperative bowel function in elderly patients with ulcerative colitis. Jpn J Gastroenterol Surg 2016;49:714–720.

[25] Kanda Y . Investigation of the freely available easy-to-use software ‘EZR’ for medical statistics. Bone Marrow Transplant 2013;48:452–458. 23208313 10.1038/bmt.2012.244 PMC3590441

[26] Tejedor P Sagias F Nock D . Advantages of using a robotic stapler in rectal cancer surgery. J Robot Surg 2020;14:365–370. 31290074 10.1007/s11701-019-00993-4

[27] Jimenez-Rodriguez RM Quezada-Diaz F Tchack M . Use of the Xi robotic platform for total abdominal colectomy: a step forward in minimally invasive colorectal surgery. Surg Endosc 2019;33:966–971. 30350106 10.1007/s00464-018-6529-x PMC6377813

[28] Fazio VW O’Riordain MG Lavery IC . Long-term functional outcome and quality of life after stapled restorative proctocolectomy. Ann Surg 1999;230:575–584; discussion 584–586. 10522727 10.1097/00000658-199910000-00013 PMC1420906

[29] Scott SM Carrington EV . The London classification: improving characterization and classification of anorectal function with anorectal manometry. Curr Gastroenterol Rep 2020;22:55. 32935278 10.1007/s11894-020-00793-z PMC7497505

[30] Liu J Guaderrama N Nager CW . Functional correlates of anal canal anatomy: puborectalis muscle and anal canal pressure. Am J Gastroenterol 2006;101:1092–1097. 16606349 10.1111/j.1572-0241.2006.00596.x

[31] Sato K Sato T . Composition and distribution of the pudendal and pelvic plexuses. Nippon Daicho Komonbyo Gakkai Zasshi 1981;34:515–529.

[32] Calomino N Martellucci J Fontani A . Care with regard to details improves the outcome of Longo mucoprolapsectomy: long term follow up. Updates Surg 2011;63:151–154. 21604057 10.1007/s13304-011-0077-4

[33] Maeda K Mimura T Yoshioka K . Japanese practice guidelines for fecal incontinence part 2-examination and conservative treatment for fecal incontinence - English version. J Anus Rectum Colon 2021;5:67–83. 33537502 10.23922/jarc.2020-079 PMC7843146

[34] Engel AF Kamm MA Bartram CI Nicholls RJ . Relationship of symptoms in faecal incontinence to specific sphincter abnormalities. Int J Colorectal Dis 1995;10:152–155. 7561433 10.1007/BF00298538

