# Supplementary material for: Comparison of short-term outcomes and defecatory function following robotic and conventional laparoscopic surgery for stapled-ileal pouch-anal anastomosis: a retrospective cohort study
Source: Int J Surg. 2024 Jul 24;110(11):7112–20. doi: 10.1097/JS9.0000000000001994 (PMC11573097; doi:10.1097/JS9.0000000000001994)
Supplement: Supplementary file 2 [file js9-110-7112-s002.pdf]

**Supplemental Figure 1.** Comparison of postoperative trends in the breakdown of Wexner score.

“Soiling” is a condition in which a stain of 3 cm or more in diameter was observed at least three times per week, whereas “spotting” is a smaller stain. Boxes indicate interquartile ranges. Lines are the median, whereas bars are the range of scores. Mean values are indicated by a cross inside the box plot.

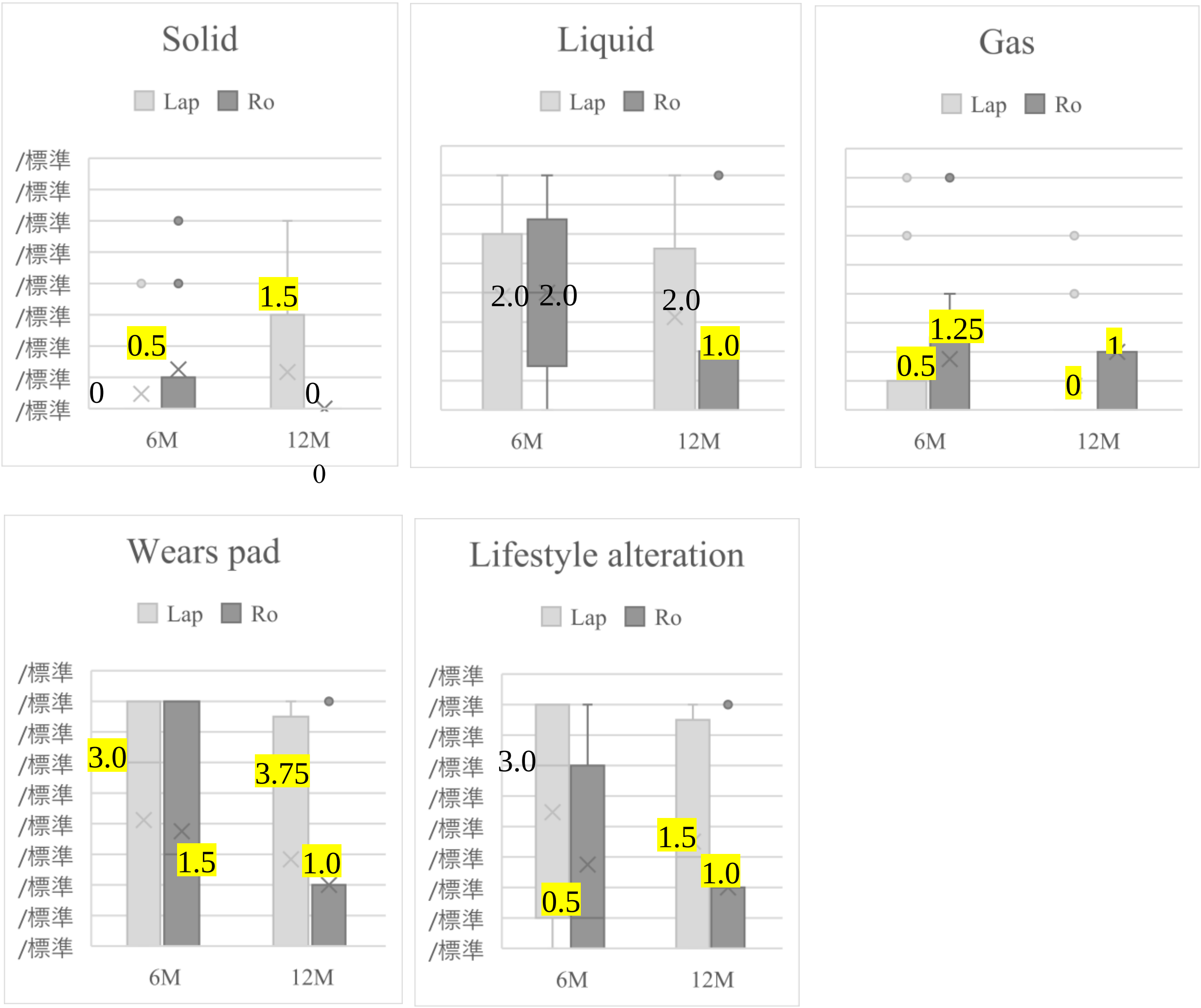

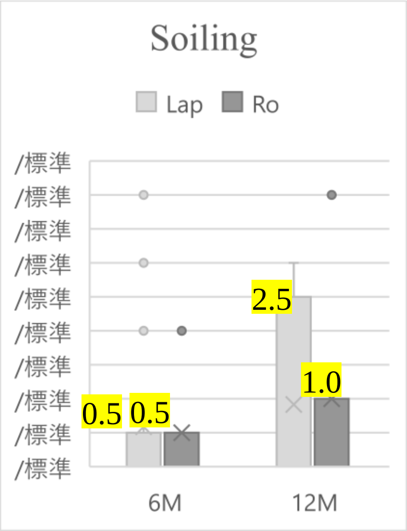

Soiling
Lap
Ro
/標準
/標準
/標準
/標準
/標準
/標準
/標準
/標準
/標準
/標準
0.5
0.5
2.5
1.0
6M
12M


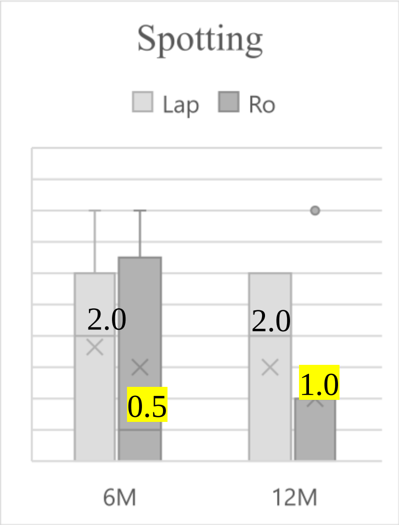

Spotting
Lap
Ro
2.0
0.5
2.0
1.0
6M
12M
